# Erythema multiforme-like targetoid eruption in two patients treated with capivasertib for metastatic breast cancer

**DOI:** 10.1016/j.jdcr.2024.09.002

**Published:** 2024-09-12

**Authors:** Heejo Keum, Jane L. Zhu, Meghan Heberton, Travis Vandergriff, Arturo R. Dominguez

**Affiliations:** a Department of Internal Medicine, University of Texas Southwestern Medical Center, Dallas, Texas; b Department of Dermatology, University of Texas Southwestern Medical Center, Dallas, Texas; c Department of Pathology, University of Texas Southwestern Medical Center, Dallas, Texas

**Keywords:** AKT inhibitor, breast cancer, capivasertib, drug reaction, erythema multiforme, targetoid

## Introduction

Capivasertib is an oral small-molecule inhibitor of the 3 protein kinase B (AKT) isoforms involved in the phosphatidylinositol 3-kinase (PI3K)–AKT–phosphatase and tensin homolog (PTEN) signaling pathway.^1^^,^^2^ Overactivation of this pathway is often found in hormone receptor (HR) positive, human epidermal growth factor receptor-2 (HER2) negative breast cancer.^3^, ^4^, ^5^ In November 2023, the US Food and Drug Administration granted approval of capivasertib for use in combination with fulvestrant for the treatment of HR-positive, HER2-negative breast cancer. In a randomized, double-blind, placebo-controlled phase III trial, CAPItello-291, rash was one of the most common adverse events of grade 3 or higher. However, these rashes are not well-characterized. This article describes two patients with targetoid eruptions mimicking erythema multiforme (EM) in patients with metastatic breast cancer after initiating capivasertib.

## Case 1

A 76-year-old woman with metastatic invasive lobular carcinoma of the breast (estrogen receptor-positive, progesterone receptor-negative, HER2-negative) presented with rash, nausea, vomiting, and diarrhea. Ten days prior, she started on capivasertib 400 mg twice daily (4-days-on, 3-days-off) and fulvestrant. She developed diarrhea soon after, requiring diphenoxylate/atropine and loperamide. During the second week of capivasertib, a pruritic rash appeared, spreading to her face, chest, abdomen, back, upper extremities, thighs, and groin.

**Fig 1:**
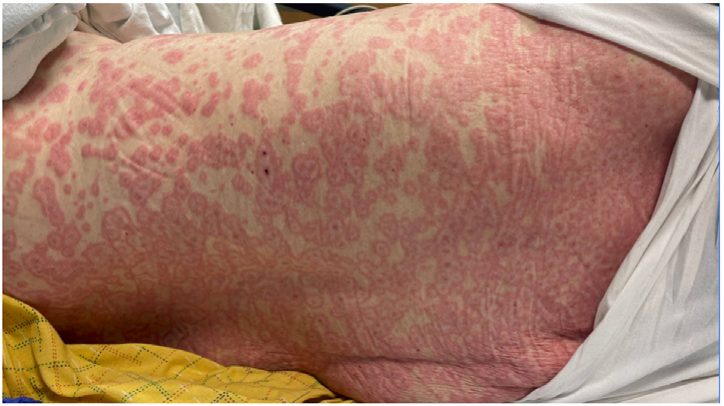
Erythematous papules and typical target lesions on the back (Case 1).

**Fig 2:**
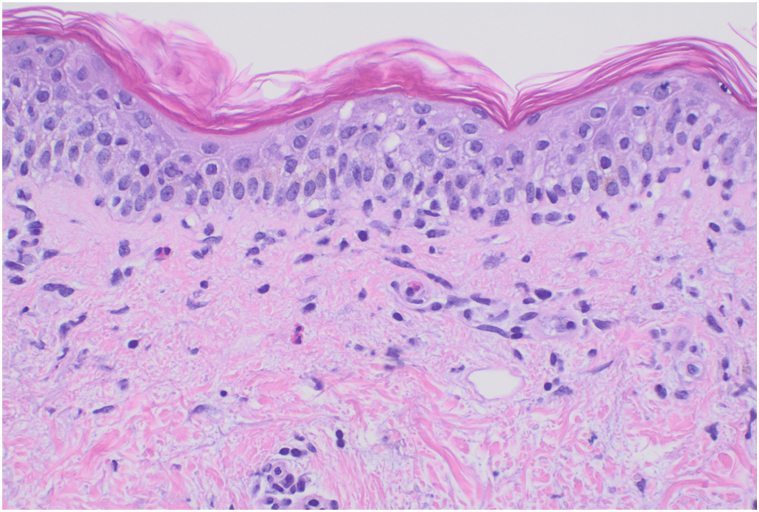
Vacuolar interface dermatitis with a superficial perivascular infiltrate of lymphocytes and a few eosinophils (Case 1).

In the emergency department, physical examination revealed numerous erythematous papules and typical targets on >30% body surface area without bullae or vesicles (Fig 1). Nikolsky sign was negative. Limited mucosal involvement was noted with a small erosion on the upper lip and vaginal introitus, and she reported significant oral pain and dysuria. Ophthalmology found no ocular involvement. She was diagnosed with a grade 3 drug eruption and received systemic steroids (one dose of intravenous (IV) methylprednisolone 1 mg/kg followed by prednisone 1 mg/kg daily) and topical steroids.^6^ Biopsies showed a superficial perivascular infiltrate of lymphocytes and eosinophils, mild vacuolar interface dermatitis, and spongiosis (Fig 2).

Her symptoms improved rapidly with treatment, and she was discharged after 3 days with instructions to continue prednisone 60 mg daily with a taper. Five days postdischarge, her lesions had resolved. An attempt to resume capivasertib at a reduced dose of 200 mg twice daily led to rash recurrence despite prednisone 20 mg daily. The rash improved with a one-time dose of prednisone 40 mg, and capivasertib was permanently discontinued.

## Case 2

**Fig 3:**
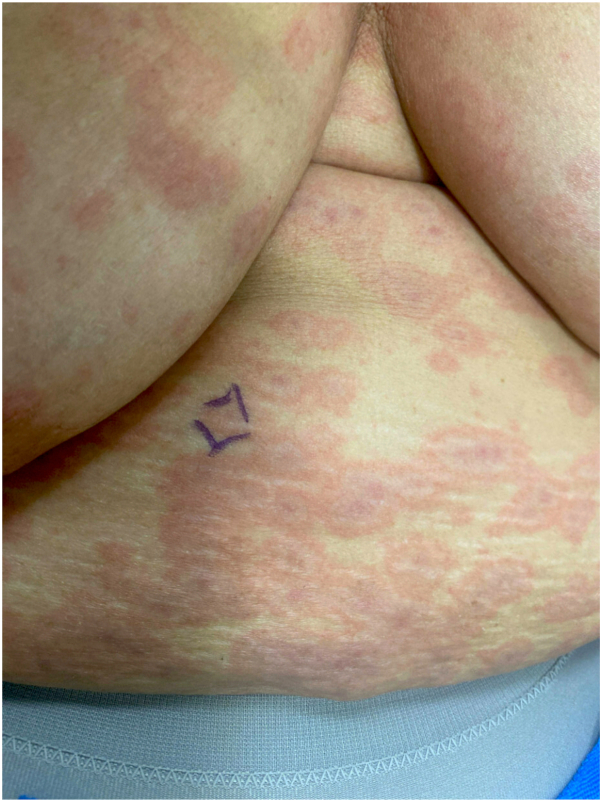
Edematous targetoid plaques and patches on the abdomen (Case 2).

**Fig 4:**
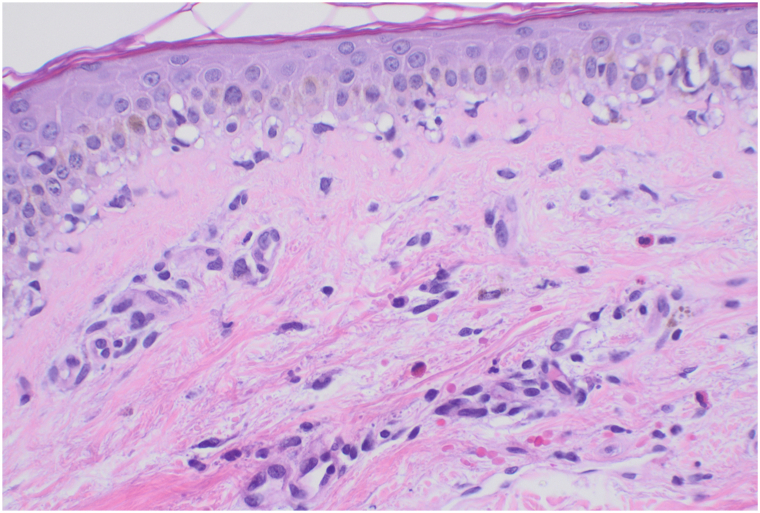
Vacuolar interface dermatitis with a superficial perivascular infiltrate of lymphocytes and eosinophils (Case 2).

A 60-year-old woman with metastatic infiltrating ductal carcinoma (estrogen receptor-positive, progesterone receptor-positive, HER2-negative) presented with a 2-day history of rash. Physical examination revealed edematous targetoid plaques on her chest, face, back, arms, legs, and palms (Fig 3). There was no mucosal involvement, and the Nikolsky sign was negative. She had started capivasertib 400 mg twice daily (4-days-on, 3-days-off) 8 days prior and developed the rash day 2 of the second week. She had received IV ceftriaxone 2g IV and levofloxacin for a urinary tract infection 2 days prior to the rash. An EM-like reaction to capivasertib was suspected, with differential diagnoses including serum sickness-like reaction to ceftriaxone, dermal hypersensitivity, and urticaria. Biopsy showed a perivascular and interstitial infiltrate of lymphocytes, neutrophils, and eosinophils, with thin epidermis and vacuolar change but no dyskeratosis (Fig 4).

Capivasertib was discontinued, and she was started on prednisone 60 mg daily and triamcinolone 0.1% cream daily. Her rash cleared within 5 days, and prednisone was tapered. Nine days after the rash resolved, the patient was restarted on capivasertib at a lower dose, 320 mg twice daily without recurrence of the rash.

## Discussion

Capivasertib combined with fulvestrant is increasingly used to treat HR-positive, HER2-negative locally advanced or metastatic breast cancer with PIK3CA/AKT1-PTEN alterations. In the phase III CAPItello-291 trial, rash occurred in 38.0% of the capivasertib–fulvestrant group versus 7.1% in the placebo–fulvestrant group, though the rash morphology was not well-described.

Our patients presented with generalized progressive eruptions with a unique morphology, clinically mimicking EM with typical and atypical targetoid lesions but minimal mucosal involvement. Biopsies revealed mild vacuolar interface dermatitis with a sparse superficial perivascular infiltrate of lymphocytes and eosinophils. Despite the focal vacuolar interface inflammation, the histologic findings of both biopsies lacked the classic features of EM, such as dyskeratotic keratinocytes, and more pronounced interface inflammation, aligning more with the histopathological findings of a morbilliform drug eruption.

Other medications targeting the PI3K–AKT–PTEN pathway have been associated with dermatological adverse effects. Approximately 40% of patients receiving alpelisib, an alpha-specific PI3K inhibitor, developed rashes, predominantly “maculopapular.”^7^ Both of our cases are similar to a recent case report describing a targetoid EM-like mucocutaneous reaction triggered by alpelisib.^8^ Additionally, idelalisib, a selective inhibitor of the delta isoform of PI3K, was associated with an EM-like reaction with mucosal involvement.^9^ The mechanism by which PI3K signaling inhibition leads to dermatologic adverse events remains unclear. It is suggested that PI3Kα signaling inhibition could induce structural changes in the epidermis and dermis due to its role in keratinocyte differentiation. Further studies are needed to determine whether these EM-like reactions stem from drug hypersensitivity, characterized by an adverse idiosyncratic immune response to the drug antigen, or are directly related to PI3K signaling inhibition as described above. Notably, unlike hypersensitivity reactions, a dose-dependent pattern was observed in our cases, with the second patient tolerating a lower dose without rash recurrence.

Following resolution of the reaction, a retrial of capivasertib at a reduced dose may be considered with close monitoring. Targeted therapies typically have dose-dependent and reproducible adverse events, as observed in our patients. It is notable that 7% of patients in the CAPItello-291 trial permanently discontinued capivasertib due to cutaneous adverse events. For patients with grade 1 reaction, it would be reasonable to rechallenge at a lower dose as is commonly done with other targeted inhibitors such as alectinib.^10^ Rechallenge with dose reduction and oral steroids can be considered based on risk-benefit assessment.

In conclusion, capivasertib and other drugs inhibiting the PI3K–AKT–PTEN signaling pathway can trigger targetoid eruptions mimicking EM. It is crucial to differentiate these eruptions from true EM and severe cutaneous adverse drug reactions such as Steven Johnson syndrome/toxic epidermal necrolysis, which can also present with targetoid lesions. In cases of true drug-induced EM or severe cutaneous adverse drug reactions, rechallenge is typically contraindicated. Given their benign clinical course with rapid response to prednisone and lack of severe mucositis, the rash was thought to be not true drug-induced EM. Our patients had drug-specific eruptions that will require further characterization over time, but as mimickers of EM, reinitiation of capivasertib at lower doses was reasonable and safe. Recognizing these polymorphic rashes in patients treated with inhibitors of the PI3K–AKT–PTEN pathway is important to avoid overtreatment and withholding of potentially life-saving drugs.

## Conflicts of interest

Dr Heberton served on an advisory board for Blueprint Medicine. Drs Keum, Zhu, Vandergriff, and Dominguez have no conflicts of interest to declare.
